# Supplementary material for: Primary Granulocyte Colony-Stimulating Factor Prophylaxis in Metastatic Pancreatic Cancer Patients Treated with FOLFIRINOX as the First-Line Treatment
Source: Cancers (Basel). 2020 Oct 27;12(11):3137. doi: 10.3390/cancers12113137 (PMC7692712; doi:10.3390/cancers12113137)
Supplement: Supplementary file 1 [file cancers-12-03137-s001.pdf]

cancers

MDPI

Article

Primary Granulocyte Colony-Stimulating Factor Prophylaxis in Metastatic Pancreatic Cancer Patients Treated with FOLFIRINOX as the First-Line Treatment

Jae Hyup Jung, Dong Woo Shin, Jaihwan Kim, Jong-Chan Lee and Jin-Hyeok Hwang

Table S1. Prophylactic G-CSF and clinical outcomes by age group.

Prophylactic primary G-CSF	<65 yr. (*N* = 102)		*p value*	≥65 yr. (*N* = 63)		*p value*
	**Yes**	No		Yes	No	
No. of patients	36/102 (35.3)	66/102 (64.7)		21/63 (33.3)	42/63 (66.7)	
Neutropenia	14/36 (38.9)	40/66 (60.6)	0.036	4/21 (19.0)	20/42 (47.6)	0.028
Febrile neutropenia	0/36 (0.0)	11/66 (16.7)	0.010	1/21 (4.8)	9/42 (21.4)	0.088
Cycles	9.0 (1–75)	6.0 (1–31)	0.062	12.0 (1–70)	6.0 (1–19)	0.031
cRDI	85.2 (47–102)	76.4 (32–109)	0.007	72.2 (43–101)	70.1 (28–101)	0.334
OS (months)	14.5 (1–73)	8.7 (2–41)	0.002	14.7 (1–65)	8.8 (0–54)	0.132

© 2020 by the authors. Licensee MDPI, Basel, Switzerland. This article is an open access article distributed under the terms and conditions of the Creative Commons Attribution (CC BY) license (http://creativecommons.org/licenses/by/4.0/).
